# Complete Genome Sequencing of *Acinetobacter* sp. Strain LoGeW2-3, Isolated from the Pellet of a White Stork, Reveals a Novel Class D Beta-Lactamase Gene

**DOI:** 10.1128/genomeA.01405-17

**Published:** 2018-01-11

**Authors:** Ulrike Blaschke, Evelyn Skiebe, Michael Kaatz, Paul G. Higgins, Yvonne Pfeifer, Gottfried Wilharm

**Affiliations:** a Robert Koch-Institut, Bereich Wernigerode, Wernigerode, Germany; b Vogelschutzwarte Storchenhof Loburg e.V., Loburg, Germany; c Institute for Medical Microbiology, Immunology and Hygiene, University of Cologne, Cologne, Germany; d German Centre for Infection Research (DZIF), Partner Site Bonn-Cologne, Cologne, Germany

## Abstract

Whole-genome sequencing of *Acinetobacter* sp. strain LoGeW2-3, isolated from the pellet of a white stork (*Ciconia ciconia*), reveals the presence of a plasmid of 179,399 bp encoding a CRISPR-Cas (clustered regularly interspaced short palindromic repeats and associated genes) system of the I-F type, and the chromosomally encoded novel class D beta-lactamase OXA-568.

## GENOME ANNOUNCEMENT

While studying the ecology of the nosocomial pathogen *Acinetobacter baumannii* (1), we isolated *Acinetobacter* sp. strain LoGeW2-3 from the pellet of a white stork (*Ciconia ciconia*) collected in Loburg, Germany, in the year 2015, following recently described protocols (2). Since partial 16S rRNA and *rpoB* gene sequencing (GenBank accession no. KT809317 and KT809318, respectively) did not indicate the strain’s belonging to any described species, PacBio RS single-molecule real-time (SMRT) sequencing was commissioned at GATC (Konstanz, Germany). Genomic DNA was isolated as recently described (3). SMRT sequencing resulted in 84,981 reads with a total of 1,145,091,354 sequenced bases and 276-fold coverage. Genome assembly using the Hierarchical Genome Assembly Process (HGAP) version 3 yielded a circular chromosome with a size of 3,178,335 bp and a circular plasmid of 179,399 bp. NCBI Prokaryotic Genome Annotation Pipeline analysis revealed a total of 3,240 genes, including 3,122 coding sequences and 118 RNA genes, of which 93 define tRNAs, as well as 7 complete rRNA gene sets and 4 noncoding RNAs. PacBio modification and motif analysis identified *N*-6-methylated adenines in motifs TGAANNNNNCTG and CAGNNNNNTTCA and an unknown modification within the motif VNCGGTGTANND (modified bases underlined).

The plasmid encodes a CRISPR-Cas (clustered regularly interspaced short palindromic repeats and associated genes) system of the I-F type (4). CRISPRDetect version 2.1 (5) identified a CRISPR array ranging from nucleotides 50488 to 45838 in reverse orientation on the plasmid and harboring 77 spacer sequences with a predominant length of 32 nucleotides. Most of the spacer sequences (61%) show highest similarity to database entries of eukaryotic origin, casting into doubt a role in targeting plasmid and phage sequences.

Twenty putative genomic islands were predicted on the chromosome by at least one method applying IslandViewer version 4 (6).

*Acinetobacter* species found in diverse environmental habitats are considered to contribute to the mobilization of antibiotic resistance genes into clinically relevant *Acinetobacter* species (7–9). A search for putative resistance genes in the genome of* Acinetobacter* sp. strain LoGeW2-3 applying ResFinder version 3.0 (10) revealed the presence of a beta-lactamase gene with an overall identity of 79% to *bla*_OXA-363_ of *Acinetobacter lwoffii*. The novel allele received the designation *bla*_OXA-568_ and the product was named class D beta-lactamase OXA-568. In its native background, no resistance phenotype could be attributed to *bla*_OXA-568_ following standard procedures, as recently described (11).

Average nucleotide identity calculations based on BLAST+ (ANIb) analysis (12) indicate that *A. schindleri* CIP 107287 is the closest relative for which whole-genome data are available (87.11% identity with 80.65% of the chromosome of *Acinetobacter* sp. strain LoGeW2-3 aligned) and support our assumption that strain LoGeW2-3 is the first representative of a novel *Acinetobacter* species.

### Accession number(s).

The complete genome sequence of *Acinetobacter* sp. strain LoGeW2-3 has been deposited at GenBank under the accession no. CP024011 (chromosome) and CP024012 (plasmid).
